# Urolithins in clinical translation: from gut microbial metabolites to precision interventions

**DOI:** 10.3389/fnut.2026.1854240

**Published:** 2026-08-06

**Authors:** Lingmin Hu, Yingchun Ling, Weifeng Jin

**Affiliations:** 1 Department of Medical Laboratory, Shaoxing Seventh People's Hospital, Affiliated Mental Health Center, Shaoxing University, Shaoxing, Zhejiang, China; 2 Department of Medical Laboratory, Shanghai Mental Health Center, Shanghai Jiao Tong University School of Medicine, Shanghai, China

**Keywords:** clinical translation, ellagitannins, gut microbiota, metabotype, mitochondrial quality control, precision nutrition, urolithin A, urolithins

## Abstract

Urolithins are gut microbiota-derived metabolites generated from ellagitannins and ellagic acid, and have emerged as promising mediators at the diet–microbiota–host interface. Interest in this metabolite family has increased substantially owing to growing evidence that urolithins, particularly urolithin A, influence mitochondrial quality control, autophagy, inflammatory signaling, and metabolic homeostasis across multiple tissues. These mechanistic properties have positioned urolithins as candidate interventions for aging-related functional decline, cardiometabolic disorders, neuroinflammatory conditions, and other chronic diseases characterized by impaired cellular resilience. However, their path to clinical application remains incompletely defined. In this Review, we synthesize current knowledge on the origin, microbial biotransformation, metabotypes, pharmacokinetics, and exposure biology of urolithins, with particular attention to the determinants of interindividual variability and the unresolved question of which molecular species are truly active *in vivo*. We then examine the core mechanisms of urolithin action and evaluate evidence across major disease contexts, integrating pre-clinical findings with available human data. Particular emphasis is placed on the current clinical literature on urolithin A, including safety, tolerability, dosing, target-engagement biomarkers, and functional endpoints. We further analyze the major factors underlying the translational gap between experimental promise and clinical outcomes, including differences in microbial production capacity, free vs. conjugated molecular forms, exposure–response uncertainty, responder heterogeneity, and limitations in trial design. Finally, we discuss how metabotype-guided stratification, microbiome-targeted strategies, improved formulations, and standardized biomarkers may support the development of urolithins within precision nutrition and translational medicine. Collectively, urolithins represent a compelling yet still evolving class of diet-derived microbial metabolites whose clinical utility will depend on resolving key mechanistic, pharmacological, and methodological uncertainties.

## 1 Introduction

Urolithins are dibenzopyran-6-one derivatives generated through the gut microbial metabolism of ellagitannins and ellagic acid, polyphenols abundant in pomegranate, walnuts, and several berries. Because the parent compounds display limited systemic bioavailability, the more absorbable downstream urolithins are increasingly regarded as the principal mediators linking ellagitannin-rich foods to host biological effects. This conceptual shift has moved urolithins from being viewed as simple catabolic by-products to being recognized as bioactive mediators at the diet–microbiota–host interface, with potential relevance to aging and chronic disease (1, 2). A defining feature of urolithin biology is marked interindividual variability. Following exposure to dietary precursors, individuals can be broadly classified into distinct urolithin metabotypes, including UM-A, UM-B, and UM-0, which reflect qualitative and quantitative differences in gut microbial conversion capacity (2). Foundational studies identified *Gordonibacter urolithinfaciens* and *Gordonibacter pamelaeae* as key bacterial species involved in ellagic acid catabolism, while subsequent work isolated additional intestinal strains capable of producing isourolithin A and further clarified why only a subset of individuals generate final urolithin metabolites (3–5). Importantly, urolithin metabotypes have been associated with host characteristics such as aging and cardiometabolic status, underscoring that exposure to bioactive urolithins depends not only on precursor intake but also on microbiome configuration (3, 4). Among the urolithin family, urolithin A has emerged as the leading translational candidate. Pre-clinical studies established urolithin A as a mitophagy activator capable of improving mitochondrial function and muscle performance in experimental models, thereby providing a strong mechanistic rationale for clinical development (5, 6). This promise has begun to extend into humans: a first-in-human study demonstrated favorable safety, bioavailability, and molecular signatures consistent with improved mitochondrial and cellular health, while a subsequent randomized clinical trial in older adults reported improved muscle endurance and favorable changes in plasma biomarkers despite neutral findings for some primary functional endpoints (7, 8). Collectively, these findings position urolithins, particularly urolithin A, at the intersection of microbiome science, healthy aging, and precision nutrition, while also highlighting the translational uncertainties that remain to be resolved (6, 9).

Accordingly, this Review is organized around the central question of how urolithins can be advanced from microbiota-dependent dietary metabolites to clinically actionable interventions. We first examine their biogenesis, metabotypes, and exposure biology, because differences in microbial conversion, conjugation, and tissue exposure are fundamental to interpreting efficacy. We then discuss the mechanistic basis of urolithin action, with emphasis on pathways most likely to be relevant *in vivo*, and critically evaluate evidence across major disease contexts by integrating pre-clinical findings with available human data. Particular attention is given to the current clinical literature on urolithin A, the translational gap between experimental promise and clinical outcomes, and the opportunities for metabotype-guided precision nutrition, improved formulation, biomarker-driven target engagement, and future therapeutic development. Although the broader urolithin family is considered throughout, the present clinical and translational evidence remains centered pre-dominantly on urolithin A (10–13). Compared with previous reviews that have comprehensively summarized urolithin metabolism, bioactivity, and associated gut microbiota (2, 14), the present Review is distinguished by three features: (i) it is organized around a single translational axis—dietary precursors, microbial conversion, *in vivo* exposure, and clinically actionable intervention—rather than cataloging bioactivities by disease; (ii) it explicitly foregrounds the exposure–activity disconnect, distinguishing physiologically achievable concentrations and conjugated species from the supraphysiologic aglycone conditions used in many mechanistic studies; and (iii) it integrates the most recent regulatory, standardization, and metabotype-stratification developments to define what is required for credible clinical translation. This framing is intended to complement, rather than duplicate, earlier metabolism- or bioactivity-centered syntheses.

## 2 Biogenesis, metabotypes, and exposure biology of urolithins

### 2.1 Dietary sources of ellagitannins and ellagic acid

Urolithin biogenesis begins with the intake of foods rich in ellagitannins (ETs) and ellagic acid (EA), but the precursor load delivered to the colon differs markedly across food sources, food forms, and processing histories. Among commonly consumed sources, pomegranate (*Punica granatum* L.) represents one of the most concentrated reservoirs of ETs, primarily as punicalagins (α and β anomers) and related gallagyl esters; in commercial pomegranate juice, the total ET-derived hydrolysis products amount to approximately 1,370 mg/L, though the ET content of the edible arils alone is far lower (~1.5 mg/g dry weight), as the majority of punicalagins reside in the husk and peels and are extracted into juice only during industrial whole-fruit processing (15). Walnuts (*Juglans regia* L.) contain pedunculagin and related ETs, with a reported total EA content after acid hydrolysis of approximately 59 mg/100 g fresh weight (1). Among berries, raspberries (*Rubus idaeus* L.) are particularly rich in sanguiin H-6 and lambertianin C, with total EA contents after hydrolysis ranging from approximately 47 to 270 mg/100 g fresh weight depending on cultivar and analytical methodology; blackberries (*Rubus* spp.) contain approximately 150 mg/100 g, and strawberries (Fragaria × ananassa) range from approximately 31 to 184 mg/100 g (1, 10), although reported values for berries differ substantially across studies due to differences in hydrolysis conditions, cultivar, and ripeness (1). Notably, even beverages such as tea can contribute meaningfully: the ET content of tea infusions, expressed after acid hydrolysis, ranges from 0.15 to 4.46 mg EA equivalents per gram of tea, corresponding to an intake of roughly 0.59 to 17.89 mg EA per cup brewed from 4 g of tea, and urolithin metabotypes A, B, and 0 have been detected in urine after tea consumption (11). Because reported precursor concentrations span a wide range across sources, cultivars, and analytical methodologies, inter-food comparisons should be based on quantitative ranges derived from standardized methods rather than single representative values (1, 12, 15).

Beyond raw content, food processing, storage, and the food matrix substantially modify the precursor actually available for colonic conversion. In processed blackberry products, canning, pureeing, and freezing caused little change in total ellagitannins, and frozen berries remained stable over six months of storage, whereas juicing led to large losses of total ellagitannins (approximately 70% for non-clarified and 82% for clarified juice) because ellagitannin-rich seeds were removed in the presscake; thermally processed products also showed compositional shifts indicative of ellagitannin depolymerization during storage (13). In addition, the food matrix can influence the absorption and downstream transformation efficiency of these compounds, for example through lipid- or phospholipid-based delivery that enhances ellagic acid bioavailability (16). Because this variability at the precursor level propagates to downstream urolithin generation, it should be explicitly considered when comparing nutritional interventions across studies (10, 11, 13, 15).

### 2.2 Gut microbial conversion to urolithins

**Figure 1:**
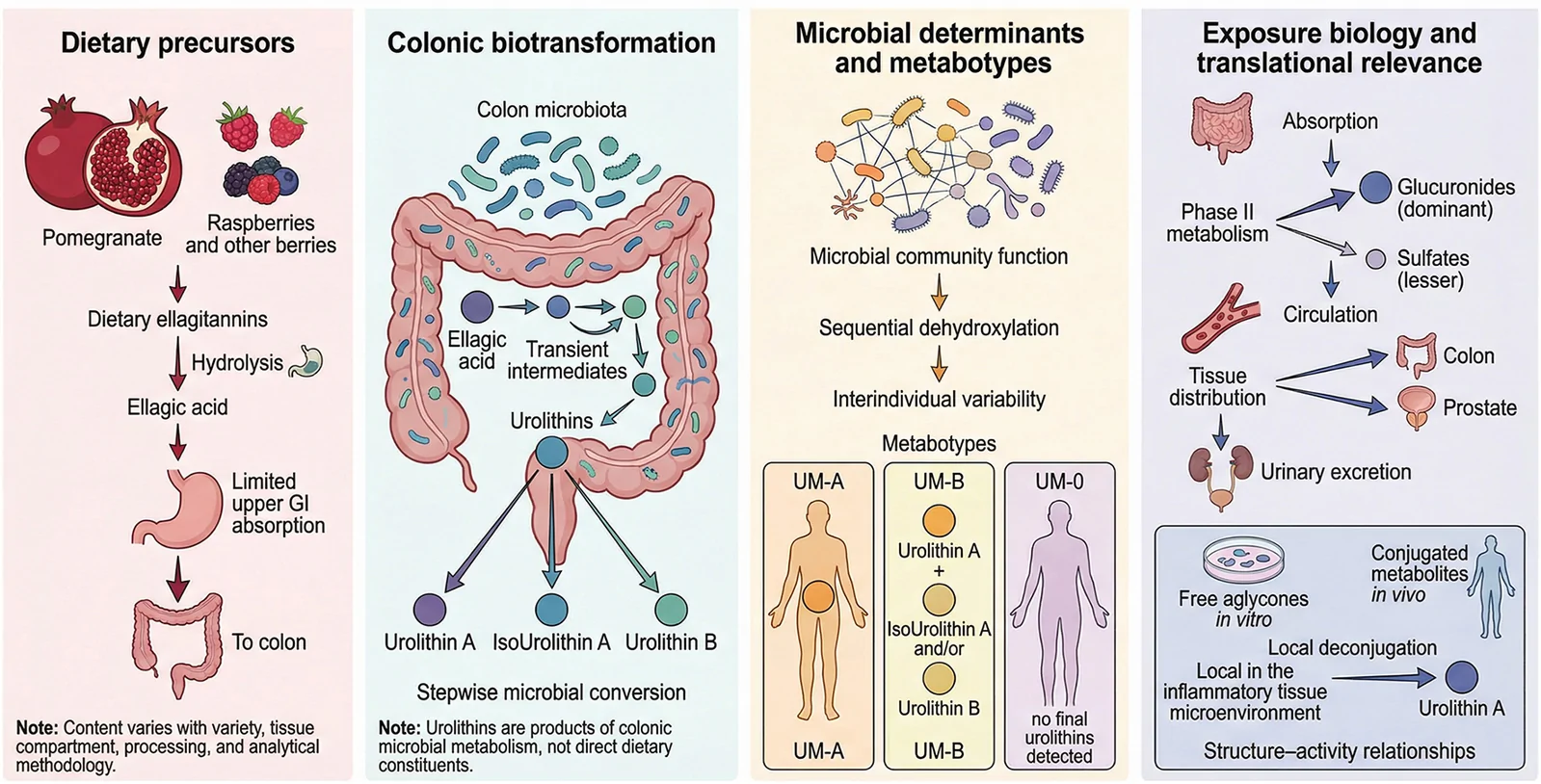
Biogenesis, metabotypes, and exposure biology of urolithins.

After ingestion, ellagitannins undergo hydrolysis to release ellagic acid, but only a limited fraction is absorbed in the upper gastrointestinal tract. A substantial proportion reaches the colon, where microbial metabolism drives the sequential conversion of ellagic acid into a spectrum of dibenzopyran-6-one derivatives collectively known as urolithins. Mechanistically, this conversion proceeds as a stepwise, regioselective dehydroxylation cascade: ellagic acid is first transformed into the pentahydroxy/tetrahydroxylated intermediates (urolithin M5 and related tetrahydroxy-urolithins), which are progressively dehydroxylated to trihydroxy-urolithins (urolithin M6 and urolithin C), and finally to the dihydroxylated end products urolithin A and isourolithin A, and the monohydroxylated urolithin B (17–19). Early human and *ex vivo* studies established that urolithins are authentic products of colonic microbial metabolism rather than direct dietary constituents, and that their appearance in plasma and urine follows delayed kinetics consistent with large-intestinal biotransformation. Time-course fermentation studies further showed that this is a chronologically ordered process in which transient hydroxylated intermediates accumulate before final metabolites, with the later dehydroxylation steps appearing comparatively slow and person-dependent, consistent with rate-limiting microbial dehydroxylase activity rather than a single fast reaction (20–23) (Figure 1).

### 2.3 Microbial determinants of urolithin formation

Urolithin production depends on the taxonomic and functional composition of the gut microbiota, and recent work allows the simple genus-level list to be reframed in functional terms. Human fecal fermentation studies first showed that the formation of intermediary and final urolithins follows person-dependent temporal patterns, indicating that microbial conversion capacity is not uniformly distributed across individuals (21). Subsequent microbiological work identified specific functional roles: *Gordonibacter* species catalyse early dehydroxylation steps toward urolithin A intermediates (24), *Ellagibacter* and related taxa are associated with isourolithin A formation (17), and *Enterocloster* species have been shown to encode the regioselective dehydroxylases that determine which urolithin end products are produced (25–27). These activities are partitioned across different organisms, so that intermediates generated by one taxon serve as substrates for another, a cross-feeding arrangement that explains why defined bacterial consortia, rather than single isolates, are typically required to reproduce complete human metabotype profiles *in vivo* (18, 19). Together, these findings support the view that sequential dehydroxylation is a distributed, division-of-labor function of the gut microbial ecosystem rather than the activity of a single organism (25–28).

### 2.4 Urolithin metabotypes and interindividual variability

A defining feature of urolithin biology is the reproducible stratification of individuals into distinct urolithin metabotypes: UM-A (urolithin A as the main final metabolite), UM-B (additional isourolithin A and/or urolithin B), and UM-0 (no final urolithins detected). Mechanistically, these phenotypes reflect the presence, abundance, and functional capacity of the dehydroxylating taxa described above, which is why defined bacterial networks can reproduce UM-A and UM-B patterns and link metabotypes to specific community functions rather than purely phenomenological readouts (18, 19, 29). Importantly, metabotype assignment is not a fixed lifelong trait but a relatively stable yet modifiable state: human studies indicate that the distribution of metabotypes is influenced by aging, and dietary pattern, disease status, and antibiotic exposure can shift conversion capacity over time (4, 30). These considerations also define the limitations of the classification, including its temporal stability, sensitivity to precursor intake and dietary background, and methodological variability in threshold definitions and sampling windows (4, 29). Despite these caveats, metabotyping has clear translational value, providing a rational basis for stratifying participants, explaining responder heterogeneity, and informing personalized nutrition and clinical trial design (4, 29).

### 2.5 Absorption, tissue distribution, metabolism, and excretion

Compared with their high-molecular-weight dietary precursors, urolithins are more readily absorbed, but their systemic disposition is dominated by extensive phase II metabolism. Human pharmacokinetic and urine studies have shown that free ellagic acid appears only transiently at low nanomolar concentrations, whereas downstream microbial metabolites are recovered pre-dominantly as glucuronide conjugates and, to a lesser extent, sulfates (22, 23, 25). Direct supplementation studies confirm that urolithin A is bioavailable in plasma across the tested dose range and that 4 weeks of oral dosing at 500 mg and 1,000 mg modulates mitochondrial biomarkers in humans (7). The magnitude and consistency of exposure differ markedly between dietary precursors and direct supplementation: after pomegranate juice intake, circulating urolithin A glucuronide reached only 110.47 ± 131.6 ng/ml at 24 h with high interindividual variability, and only ~40% of subjects were high converters, whereas a single 500 mg dose of direct urolithin A produced a peak plasma urolithin A glucuronide of 480.75 ± 238.03 ng/ml at 6 h, declining to 255.52 ± 129 ng/ml at 24 h, thereby achieving more uniform exposure across the population (26). Reported pharmacokinetic parameters (Cmax, Tmax, AUC, and the pre-dominance of conjugated species) should be summarized comparatively for dietary vs. direct-supplement exposure (Table 1). For dietary precursors, plasma free ellagic acid is low and highly variable (Cmax 74.8 ± 54.4 nm and 64.1 ± 76.8 nm for two pomegranate extracts; Tmax ~1.5–2 h; AUCall 0.25–0.42 μm·h) and rarely exceeds 100 nm, with urolithins appearing in plasma only after 24 h (23); by contrast, direct urolithin A supplementation (250–1,000 mg) produces dose-dependent increases in Cmax and AUC, with Tmax at 6 h and elimination half-lives of 17–22 h for urolithin A and its glucuronide and 25–58 h for the sulfate (7). Tissue-distribution studies further indicate that ellagitannin-derived metabolites, including urolithins, can be detected in human target tissues such as the prostate and colon after dietary exposure or pomegranate extract supplementation (27, 28). Critically, however, detection of urolithins in tissue does not by itself establish that biologically effective concentrations are reached, because the species detected are largely conjugated and present at low concentrations; physiologically-based pharmacokinetic modeling indicates that the concentrations associated with either beneficial or toxic effects *in vitro* are unlikely to be achieved *in vivo* (31). Exposure biology must therefore be interpreted across multiple levels, including precursor release, microbial conversion efficiency, intestinal absorption, hepatic and enterocytic conjugation, tissue delivery, and urinary elimination (29, 30, 32–35).

**Table 1:** Human pharmacokinetic parameters for dietary ellagic acid/urolithins vs. direct urolithin A.

Analyte (source)	Dose	Cmax	Tmax	AUC	t$\frac{1}{2}$/notes	References
Free ellagic acid (PE-1)	1.8 g extract (524 mg EA)	74.8 ± 54.4 nm (median 67; 12–193)	1.97 ± 1.85 h (median 1)	0.42 ± 0.67 μm·h (0.01–2.73)	Plasma EA rarely >100 nm	(26)
Free ellagic acid (PE-2)	1.8 g extract	64.1 ± 76.8 nm (median 37; 15–360)	1.42 ± 1.08 h (median 1)	0.25 ± 0.39 μm·h (0.01–1.69)	High interindividual variability	(26)
Urolithins (dietary, plasma)	via PE intake	IsoUro-A glur 153 ± 107 nm; Uro-B glur 28 ± 17 nm (UM-B only)	Detected only >24 h	—	Appear late; producer-dependent	(26)
Urolithin A (direct supplement)	250–1,000 mg	Dose-dependent ↑ (values in Figure 1)	6 h	Dose-dependent ↑	t$\frac{1}{2}$: UA & UA-gluc 17–22 h; UA-sulfate 25–58 h	(10)

### 2.6 Free vs. conjugated urolithins: implications for *in vivo* activity

A central translational challenge is that the dominant molecular species present *in vivo* are usually conjugated metabolites, whereas many mechanistic studies have tested free aglycones. Human urine analyses have chemically characterized major urolithin glucuronides, underscoring that conjugation is a defining feature of systemic exposure (25). Functional comparisons indicate that glucuronidation can substantially attenuate bioactivity: in human and murine immune-cell models, urolithin A was the most active metabolite (for example, ~91% inhibition of the LPS-induced inflammatory response at 40 μm), whereas none of the corresponding glucuronide conjugates inhibited TNF-α or induced IL-10, indicating that glucuronidation can substantially reduce pharmacological activity, at least in immune-cell models (32). However, this loss of activity is not absolute: in human aortic endothelial cells, urolithin A glucuronide retained anti-inflammatory effects, and urolithin B-glucuronide showed activity on nitric oxide-related responses, suggesting that the functional consequences of conjugation depend on both the urolithin scaffold and the target cell type (33, 34). These cell-type-dependent differences indicate that the activity of conjugated urolithins should not be generalized across all biological contexts, and that the translational relevance of glucuronides may vary by tissue and indication (32–34). The frequently invoked mechanism of local tissue deconjugation, whereby beta-glucuronidase activity at inflamed sites regenerates free urolithin A, offers a plausible reconciliation of low circulating free concentrations with tissue-level activity. It is important to state, however, that current support for this mechanism is largely indirect and derived from *in vitro* or *ex vivo* observations, and that its quantitative occurrence in humans *in vivo* has not been directly demonstrated (25, 32, 35). This distinction should be made explicit to avoid overstating the certainty of local reactivation.

### 2.7 Structural diversity and structure–activity relationships

The urolithin pathway generates a broader chemical landscape than is implied by the pre-dominant focus on urolithin A, and the functional differences among variants can be organized within a systematic structure-activity framework. Novel intermediates and minor metabolites continue to be identified in human feces and urine, reflecting multiple hydroxylation patterns and transitional structures (36). The number and position of hydroxyl groups govern lipophilicity, membrane permeability, target engagement, and susceptibility to phase II conjugation, which together determine metabolic stability and cellular activity. In comparative studies, urolithin A frequently shows stronger activity than isourolithin A, urolithin B, or their conjugated derivatives in selected inflammatory and senescence-related models (32), while other end products display their own activity profiles: urolithin C suppresses colorectal cancer progression via a YBX1-AKT/mTOR axis (37) and blocks NF-κB signaling in LPS-stimulated macrophages (38), and urolithin D has been reported to counter oxidative stress (39), illustrating that activity is variant- and context-specific rather than confined to urolithin A. A concise SAR table relating hydroxylation pattern, predicted lipophilicity, conjugation propensity, and reported activity would make these relationships explicit (Table 2; values from PubChem and cited studies). Accordingly, structure-activity relationships should not be reduced to a ranking of canonical end products, but considered within the broader framework of metabolite mixtures, local deconjugation, and tissue-specific exposure (32, 36, 40).

**Table 2:** Structural features, conjugated metabolites, and reported bioactivities of representative urolithins.

Urolithin	Structural feature	Major conjugates in humans	Main matrices	Reported bioactivities	XLogP3 (PubChem)	Translational relevance
Urolithin A (UA)	Dihydroxy urolithin; most extensively studied member	Mainly glucuronides; sulfates also reported; free aglycone usually minor	Plasma, urine, selected tissue-related readouts	Mitophagy activation, improved mitochondrial function, anti-inflammatory effects, muscle/endurance-related benefits, cardiometabolic and vascular biomarker modulation (10, 11, 14, 44, 58)	2.3	Lead translational candidate with the strongest evidence for safety, bioavailability, target engagement, and functional benefit (10, 11, 13–15)
Iso-urolithin A (iso-UA)	Positional isomer of UA	Conjugated forms expected, especially glucuronides; less characterized clinically than UA	Urine, plasma	Distinct antiproliferative and metabolic effects compared with UA, supporting non-equivalent structure-activity relationships (80)	2.3	Relevant for SAR analysis, but direct clinical evidence remains limited
Urolithin B (UB)	Monohydroxy downstream urolithin	Glucuronides and sulfates reported	Plasma, urine	Anti-inflammatory effects, endothelial nitric oxide-related activity, and cartilage-protective effects in pre-clinical models (46, 78, 79)	2.7	Major microbial end product with translational relevance, but much less clinically developed than UA
Urolithin C (UC)	Trihydroxy intermediate/product in the conversion pathway	Phase II conjugates plausible, but less emphasized in translational studies	Mainly urine/feces, pathway-mapping studies	Broad antioxidant and anti-inflammatory activities in mechanistic and pre-clinical literature (12)	2.0	Useful as a pathway/comparator metabolite, but not a leading clinical candidate
Urolithin D (UD)	Tetrahydroxy urolithin closer to upstream intermediates	Human circulating conjugates insufficiently characterized	Mainly metabolite-mapping studies	Primarily biosynthetic relevance rather than established intervention evidence (12)	1.6	Best regarded as a biotransformation intermediate
Other upstream intermediates	Early dehydroxylation intermediates during ellagitannin/ellagic acid conversion	Limited human data	Mostly feces or experimental microbial systems	Mainly pathway relevance with limited direct functional evidence	1.3 (Uro-M5)	Can be noted for pathway completeness without shifting the focus from downstream urolithins

## 3 Mechanistic basis of urolithin action

### 3.1 Mitochondrial quality control and bioenergetics

A central mechanistic theme in urolithin research is the regulation of mitochondrial quality control, but three related yet distinct concepts must be clearly separated: mitophagy (selective autophagic removal of damaged mitochondria), mitochondrial biogenesis (generation of new mitochondria), and overall improvement of mitochondrial function (e.g., respiration, ATP output, membrane potential). These are not interchangeable, and the strength of evidence differs across readouts: direct demonstration of mitophagy requires reporter-based or flux-blocking approaches and documentation of PINK1-PARKIN recruitment, whereas changes in respiration or reactive oxygen species are indirect indicators of function rather than proof of mitophagy activation. In UVA-injured human dermal fibroblasts, urolithin A attenuated oxidative damage, improved mitochondrial homeostasis, and promoted mitophagy in association with the SIRT3-FOXO3-PINK1-PARKIN axis (41). Because these data are largely correlative, it cannot yet be resolved whether modulation of this axis is a direct effect of urolithin A or a secondary consequence of reduced oxidative stress and improved cellular status; this should be stated explicitly, and causal attribution awaits loss-of-function or upstream-intervention experiments. Consistency across models is also incomplete: urolithin A improved cardiac function with enhanced mitochondrial quality in heart failure settings (42), whereas in an early Alzheimer disease cellular model it mainly induced transcriptional responses linked to mitochondrial biogenesis and oxidative phosphorylation with limited improvement in respiration, suggesting hormetic or model-specific effects (43).

### 3.2 Inflammatory and redox signaling

**Figure 2:**
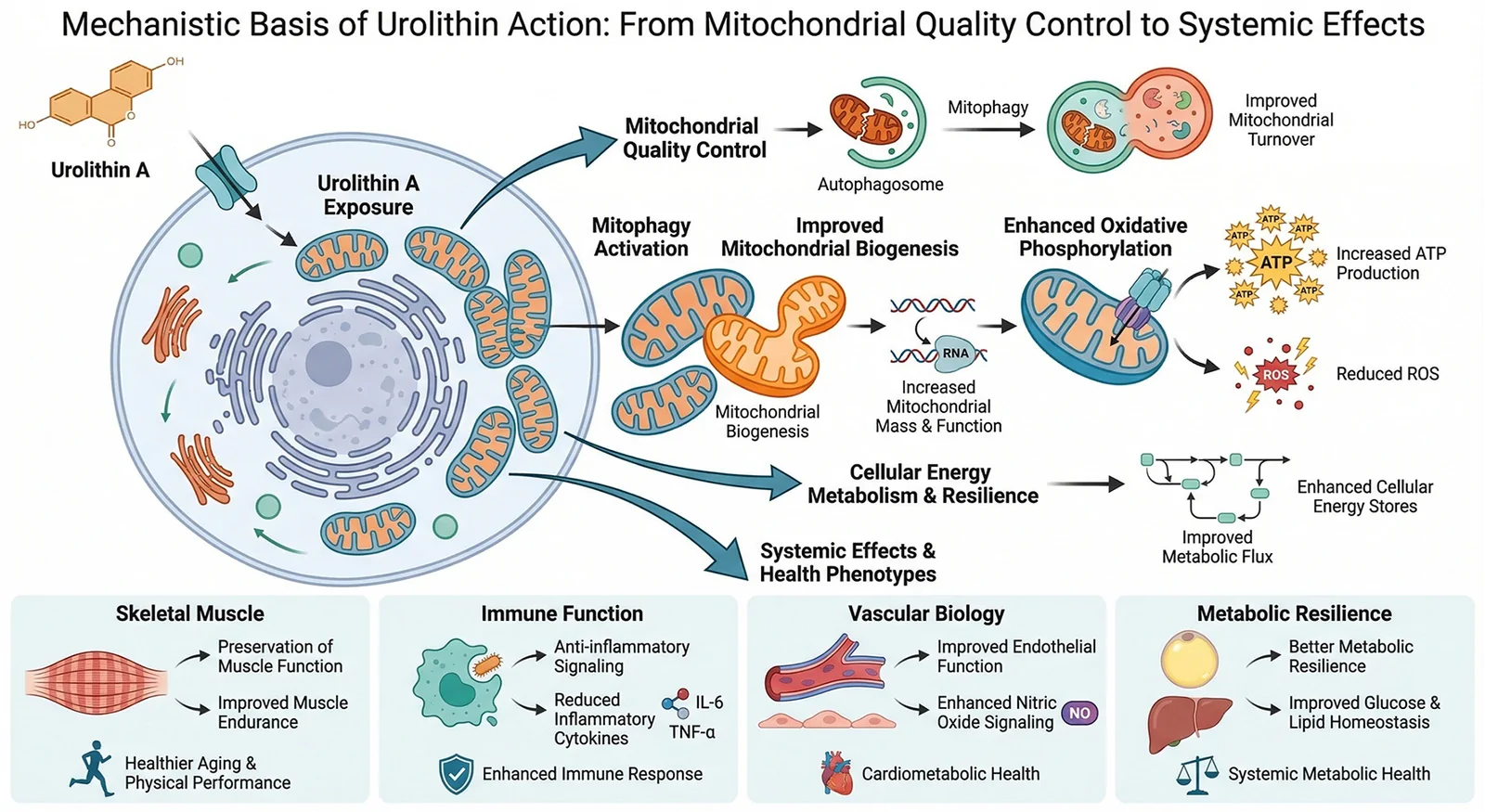
Mechanistic basis of urolithin action *in vivo*.

Beyond mitochondrial turnover, urolithin A modulates a connected inflammatory signaling network rather than a set of independent pathways. In lipopolysaccharide-stimulated macrophages, it suppressed PI3K/Akt/NF-kB and JNK/AP-1 activation and reduced NADPH oxidase-derived reactive oxygen species, with NADPH oxidase-derived ROS acting as a shared upstream node that feeds into both the NF-kB and JNK/AP-1 arms, thereby attenuating pro-inflammatory mediator production (44). These pathways are further integrated with intracellular quality-control machinery: urolithin A enhances autophagic flux in macrophages, and this autophagy response contributes directly to reduced p65 nuclear translocation and diminished M1 polarization, functionally linking mitophagy/autophagy to inflammatory signaling (45). The interplay is bidirectional, because mitochondrial ROS can activate NF-kB while improved mitochondrial quality lowers the oxidative tone that drives these cascades. These cell-based observations are supported *in vivo*, as orally administered urolithin A reduced carrageenan-induced paw edema and increased plasma antioxidant capacity in mice (46). These interconnected redox, NF-κB/JNK, and autophagy nodes are summarized in the mechanistic overview (Figure 2).

### 3.3 Barrier-protective and endothelial actions

In intestinal epithelial models, urolithin A protected against TNFα-induced barrier dysfunction in HT-29/B6 cells by inhibiting the inflammation-driven upregulation of claudin-1 and claudin-2 and by correcting claudin-1 delocalization, thereby preserving junctional organization; ellagic acid acted through a partially distinct, claudin-modulating mechanism (downregulation of claudin-4,−7 and−15 via myosin light chain kinase) and was most effective at strengthening the barrier *per se* in ileum-like Caco-2 cells, increasing transepithelial resistance and reducing paracellular permeability (47). Notably, the tight junction scaffolding protein zonula occludens-1 (ZO-1) and the transmembrane sealing protein occludin—two additional components essential for barrier integrity—were not assessed in that study. In a mouse model of hexavalent chromium [Cr (VI)]-induced small intestinal toxicity, urolithin A co-administration (20 mg/kg for 28 days) partially restored occludin protein expression that had been reduced by Cr (VI) exposure (9.43–44.60% decrease in wild-type mice), and attenuated the Cr (VI)-induced increase in intestinal permeability by 44.7% (48). In the same model, Cr (VI) also disrupted ZO-1 expression and distribution; however, the effect of urolithin A on ZO-1 was documented in supplementary data rather than quantified in the primary analysis (48). The protective effect of urolithin A in this model was attributed to modulation of the PP2A/Hippo/YAP1 signaling pathway rather than to direct engagement of tight junction regulatory cascades (48). Collectively, the available evidence indicates that urolithin A can preserve multiple tight junction components beyond claudins, including occludin, but direct, quantitative evidence for ZO-1 regulation by urolithins in intestinal epithelium remains limited and warrants further investigation. These findings indicate that precursor and microbial metabolite engage different tight-junction targets, with ellagic acid acting preventively and urolithin A protecting against inflammation-induced barrier loss (47). In colitis models, urolithin A-mediated protection also requires host xenobiotic-response signaling, as CYP1A1 was essential for its anti-inflammatory and barrier-protective effects (49), and barrier integrity has additionally been linked to Nrf2-dependent mechanisms (50). In the vascular compartment, both urolithin A and its glucuronide ameliorated TNF-α-induced inflammatory changes in human aortic endothelial cells, suggesting that even conjugated metabolites may retain functional relevance in selected inflammatory microenvironments (33).

### 3.4 Senescence, tissue remodeling, and stress adaptation

The downstream consequences of these pathways include effects on senescence-associated phenotypes and tissue remodeling, and these outcomes are strongly context dependent. In replicative senescent human skin fibroblasts, urolithin A increased type I collagen expression, reduced matrix metalloproteinase-1, and lowered intracellular reactive oxygen species, consistent with a shift toward a less degradative and less stress-burdened cellular state (51). In UVA-induced photoaging models, these effects were accompanied by attenuation of senescence-associated features and restoration of mitochondrial homeostasis (41). However, the effect of urolithin A on senescence is not uniformly suppressive across all biological settings. In human colon cancer cells, prolonged exposure induced a p53-dependent senescence program rather than simply reversing senescence markers, highlighting that urolithin A can either restrain or promote senescence depending on cell type, exposure conditions, and disease context (52).

### 3.5 Cell cycle control and anticancer mechanisms

These observations point to a broader principle: the actions of urolithin A are governed by four interacting determinants—cell type, disease background, dose range, and treatment duration—rather than being uniformly protective. In androgen-independent prostate cancer cells, urolithin A contributed to growth suppression through effects on cell cycle control and apoptosis, particularly when considered together with ellagic acid in physiologically relevant metabolite combinations (53). In colorectal cancer cells, urolithin A induced dose- and time-dependent growth inhibition accompanied by cell cycle arrest, increased p53 and p21 expression, suppression of Bcl-2, cytochrome c release, caspase activation, and reactive oxygen species accumulation (54). Together with its ability to trigger p53-dependent senescence in selected colorectal cancer models (52), these findings indicate that the anticancer effects of urolithin A emerge from coordinated modulation of redox balance, mitochondrial signaling, cell cycle checkpoints, and stress-response programs rather than a single dominant pathway (53–55). Overlaying these determinants is the question of physiological relevance: many *in vitro* studies use micromolar concentrations, yet free systemic urolithin A is present largely as low-concentration conjugates, and PBPK modeling indicates that the concentrations producing effects *in vitro* are unlikely to be achieved *in vivo* (31); mechanistic conclusions drawn from supra-physiological exposures should therefore be interpreted with explicit caution regarding human relevance (Figure 2).

## 4 Urolithins across disease contexts: pre-clinical evidence and human relevance

Consistent with the translational scope and title of this Review, the following disease-context subsections summarize the most informative pre-clinical evidence in condensed form and place greater emphasis on human relevance and translational challenges; the detailed human intervention literature is consolidated and critically appraised in Section 5 (see also Table 3).

**Table 3:** Summary of published human studies of urolithin interventions.

Study	Population	Intervention	Duration	Main readouts	Key findings	Translational interpretation	Major limitations	References
Andreux et al.	Healthy older adults in a first-in-human setting	Single and repeated oral UA dosing	Up to 28 days	Safety, tolerability, plasma exposure, metabolomics, skeletal muscle molecular readouts	UA was safe and bioavailable and induced plasma and muscle signatures consistent with improved mitochondrial and cellular health	Best interpreted as a phase 1 target-engagement study, not an efficacy trial	Small; short ( ≤ 28 d); safety/biomarker endpoints, not clinical efficacy	(10)
Singh et al.	Healthy adults	Direct UA supplementation compared with dietary precursor exposure	Short-term exposure study	Exposure consistency, producer vs. non-producer differences, interindividual variability	Direct UA supplementation produced more consistent exposure than microbiota-dependent conversion from dietary precursors	Supports the precision-nutrition rationale and the use of direct supplementation to reduce exposure variability	Exposure (not clinical) endpoint; short-term; healthy adults	(13)
Liu et al.	Older adults	UA supplementation vs. placebo	Randomized trial	Muscle endurance, physical performance, plasma biomarkers	UA improved hand and leg muscle endurance and favorably changed plasma biomarkers linked to mitochondrial health, while some broader functional endpoints remained unchanged	Highlights the importance of endpoint hierarchy; muscle/endurance and biomarker outcomes may be more sensitive than broad functional measures	Primary endpoints (6-min walk, ATP) neutral; modest sample	(11)
Singh et al.	Middle-aged adults	Randomized UA supplementation trial	Randomized trial	Muscle strength, exercise performance, biomarkers of mitochondrial health	UA improved muscle strength, exercise performance, and biomarkers related to mitochondrial function	Provides proof-of-concept for biomarker-linked functional translation in an at-risk but non-diseased population	Primary endpoint (peak power) neutral; relatively healthy population	(14)
Liu et al.	Human cardiovascular context	UA intervention with cardiovascular biomarker assessment	Human translational study	Cardiovascular health biomarkers	Improvement in cardiovascular-related biomarkers was reported alongside pre-clinical cardioprotective observations	Suggests cardiometabolic relevance, but evidence remains biomarker-level rather than disease-outcome level	Early-stage human signal; limited sample; surrogate biomarkers	(44)
Whitfield et al.	Highly trained male distance runners during altitude training	UA supplementation	4 weeks	3,000 m time trial, perceived exertion, muscle damage markers, skeletal muscle proteomics	No significant improvement in time-trial performance, but perceived exertion decreased, indirect muscle damage markers were attenuated, and mitochondrial-related proteomic changes were observed	Illustrates partial translation: molecular adaptation without clear performance gain in a high-functioning population	No significant performance benefit; small, highly trained cohort	(56)
Monsalve Acevedo et al.	Academy soccer players during preseason	UA supplementation	6 weeks	Intermittent running, jump performance, antioxidant/performance-related outcomes	Selected intermittent running and jumping outcomes improved, whereas several endpoints remained neutral	Suggests possible benefit in sports-performance settings, but evidence remains preliminary and pilot-scale	Small sample; selected endpoints only; short duration	(57)

### 4.1 Musculoskeletal aging, sarcopenia, and exercise adaptation

Musculoskeletal aging remains one of the clearest areas in which urolithin research has moved from mechanistic plausibility toward translational relevance. In highly trained male distance runners, 4 weeks of urolithin A supplementation during altitude training did not improve 3,000 m time-trial performance, but it reduced perceived exertion, attenuated indirect markers of muscle damage, and shifted skeletal muscle proteomic signatures toward mitochondrial pathways (55). In academy soccer players, 6 weeks of supplementation during pre-season improved selected intermittent running and jumping outcomes, although the study was small and several endpoints remained neutral (56). These athlete-based studies suggest that urolithin A may be more relevant to recovery and adaptation than to universal enhancement of competitive performance itself. In humans, the strongest randomized evidence is in this musculoskeletal domain: urolithin A improved muscle endurance, strength (by ~12% in one trial), and mitochondrial biomarkers, yet several pre-specified primary endpoints (6-min walk distance, maximal ATP production, and peak power output) were not significantly improved, underscoring a translational gap between mechanism and clinical benefit (8, 57).

The pre-clinical musculoskeletal literature is broader and mechanistically more coherent. In experimental models of muscular dystrophy, urolithin A restored mitophagy, increased skeletal muscle respiratory capacity, improved muscle stem-cell regenerative ability, and improved muscle function and survival, supporting the view that mitochondrial quality control is directly relevant to muscle integrity under degenerative stress (58). The tissue scope of musculoskeletal benefit also extends beyond skeletal muscle. In osteoarthritis models, urolithin A improved mitophagy and mitochondrial respiration in primary human chondrocytes derived from both healthy donors and patients with osteoarthritis, and *in vivo* reduced cartilage degeneration, synovial inflammation, and pain (59). Taken together, these studies support a disease-context model in which urolithin A is most plausibly linked to fatigue resistance, tissue resilience, and musculoskeletal recovery rather than to a single performance phenotype.

### 4.2 Obesity, insulin resistance, and metabolic dysfunction-associated steatotic liver disease

Metabolic dysfunction is another major area in which urolithins show strong pre-clinical relevance. In mice with diet-induced insulin resistance, ellagic acid and its microbial metabolite urolithin A improved glucose homeostasis and insulin sensitivity, indicating that conversion to downstream gut-derived metabolites contributes materially to the metabolic effects of ellagitannin-rich foods (60). In obesity models, urolithin A reduced weight gain and metabolic dysfunction by increasing energy expenditure, enhancing brown adipose tissue thermogenesis, and promoting browning of white adipose depots (61). These findings suggest that urolithin A can act not only on glucose handling but also on systemic energy balance.

This metabolic relevance also extends to hepatic disease phenotypes now encompassed within metabolic dysfunction-associated steatotic liver disease. In fructose-driven hepatosteatosis, urolithin A reduced hepatic lipid accumulation, suppressed lipogenic reprogramming, enhanced β-oxidation, and induced lipophagy (62). Accordingly, the current evidence supports urolithin A as a candidate modulator of interconnected metabolic phenotypes spanning insulin resistance, obesity, and steatotic liver injury (60–62). However, direct human efficacy data in these disease populations remain limited, so the present case is still driven mainly by pre-clinical data rather than disease-specific trials.

### 4.3 Neuroinflammation, neuroprotection, and cognitive aging

The neurological literature suggests that urolithin A acts across several overlapping pathogenic axes relevant to aging brain disorders. In APP/PS1 mice, urolithin A attenuated memory impairment, reduced amyloid-β deposition, decreased microgliosis and astrocytosis, and improved neurogenesis and neuronal survival (63). In Parkinson disease models, it promoted mitophagy and suppressed NLRP3 inflammasome activation in both microglial cells and MPTP-treated animals, linking mitochondrial quality control to reduced neuroinflammatory signaling (64). In a separate Alzheimer disease model, long-term intermittent supplementation reduced amyloid-β burden and improved multiple behavioral outcomes, further supporting a neuroprotective profile *in vivo* (65). Human evidence in the neurocognitive domain remains preliminary and is largely limited to ongoing trials with cognition-related endpoints, so neuroprotective claims should currently be regarded as hypothesis-generating rather than clinically established.

The relevance of urolithin A may also extend to metabolically stressed neuronal states. In neuronal models exposed to high glucose, urolithin A suppressed amyloidogenic signaling by modulating TGM2-dependent endoplasmic reticulum–mitochondria contacts, calcium dysregulation, and mitochondrial reactive oxygen species accumulation (66). Together, these studies suggest that the neurological significance of urolithin A does not depend on one isolated pathway, but rather on convergent regulation of neuroinflammation, mitochondrial dysfunction, proteopathic stress, and metabolic vulnerability.

### 4.4 Inflammaging and immune dysregulation

The concept of inflammaging provides an especially useful framework for interpreting emerging urolithin data. In a randomized placebo-controlled trial in healthy middle-aged adults, short-term oral urolithin A supplementation remodeled immune-cell composition and metabolism toward a less age-associated dysfunctional profile, supporting the view that mitophagy induction can translate into measurable benefits in human immune aging (67). This is important because it moves urolithin biology beyond muscle-centered outcomes and into a broader host-defense context.

Pre-clinical evidence supports this interpretation. Urolithin A expanded stem-like memory T cells through mitophagy induction and enhanced antitumor immunity *in vivo*, indicating that its immune effects are not limited to dampening inflammation but can also improve adaptive immune fitness (68). In another inflammatory setting, urolithin A suppressed NLRP3 inflammasome activation, reduced IL-1β maturation and pyroptosis, and prevented monosodium urate crystal-induced peritonitis, showing that its immunomodulatory role also extends to innate inflammatory signaling (69). Collectively, these findings make inflammaging and immune dysregulation one of the strongest bridges between urolithin mechanisms and human relevance.

### 4.5 Cardiovascular and endothelial dysfunction

Cardiovascular relevance has emerged through endothelial, myocardial, and vascular-inflammation models. In a placebo-controlled human trial, the effect of urolithin A on vascular endothelial function depended on baseline gut microbiota characteristics, emphasizing that cardiovascular responsiveness may be conditioned by host–microbiome context rather than by supplementation alone (70). This human signal is still preliminary, but it is important because it aligns vascular physiology with the broader concept of responder heterogeneity. Overall, human cardiovascular data are still early-stage and context-dependent, reinforcing that translational confidence requires confirmation in adequately powered, endpoint-driven trials.

Pre-clinical work suggests that the cardiovascular scope of urolithin A is wider than endothelial function alone. In myocardial ischemia–reperfusion injury, urolithin A improved cardiac function, attenuated oxidative stress and ferroptosis, and activated Nrf2-dependent antioxidant defenses (71). In ApoE-deficient mice, urolithin A decreased atherosclerotic lesion burden, reduced endothelial inflammatory activation and plaque macrophage content, and increased fibrous-cap features associated with plaque stabilization (72). Together, these findings support cardiovascular and endothelial dysfunction as plausible translational targets, although definitive clinical endpoint data are still lacking.

### 4.6 Intestinal barrier dysfunction and related conditions

Because urolithins are generated in the colon and extensively exposed to the intestinal mucosa, barrier dysfunction and intestinal inflammation are particularly plausible therapeutic contexts. Urolithin A has been shown to enhance gut barrier integrity through Nrf2-dependent mechanisms and to mitigate inflammation and colitis in pre-clinical systems (50). Earlier work in experimental colitis also showed that a pomegranate extract and its metabolite urolithin A exert anti-inflammatory effects in the colon, while simultaneously demonstrating that intestinal inflammation itself can alter phenolic metabolism (73). These findings are conceptually important because they suggest a bidirectional relationship between intestinal disease and urolithin exposure biology (50, 73).

More recent studies have refined this intestinal disease framework. In dextran sulfate sodium-induced colitis, urolithin A alleviated disease severity while improving gut microbiota dysbiosis, modulating microbial tryptophan metabolism, and triggering AhR activation (74). In infectious colitis models, urolithin A also reduced ^*^Clostridioides difficile^*^ toxin expression and attenuated toxin-induced epithelial damage, suggesting that it may act at both the host-barrier and pathogen-virulence levels (75). Overall, the gastrointestinal field provides one of the most coherent disease-context rationales for urolithin biology, even though controlled human intervention data are still sparse.

### 4.7 Beyond urolithin a: functional specificities of other urolithins

Although urolithin A dominates the translational literature, other urolithins are not functionally redundant. Urolithin B has been identified as a regulator of skeletal muscle mass, promoting hypertrophy and attenuating atrophy in experimental models (76). In activated microglia, urolithin B also exerted anti-inflammatory and antioxidant effects through modulation of multiple signaling pathways, supporting potential relevance in neuroinflammatory settings (77). These studies indicate that biologically meaningful activity is not exclusive to urolithin A.

Functional divergence also appears in vascular and joint models. In human aortic endothelial cells, urolithin B and urolithin B-glucuronide influenced nitric oxide biology and endothelial NO synthase-related responses, suggesting that endothelial activity is shared across at least some non-urolithin-A metabolites (34). In osteoarthritis models, urolithin B reduced cartilage degeneration and alleviated inflammation-driven joint damage (78). In addition, isourolithin A has shown distinct antiproliferative activity and a profile that is not identical to its urolithin A isomer, indicating that subtle structural variation can translate into functional non-equivalence (79). Therefore, future disease-oriented work should not assume that all biological relevance resides in urolithin A alone; rather, the broader urolithin family may contain metabolite-specific activities that are currently underexplored (Table 2).

## 5 Clinical evidence and the translational gap

### 5.1 Human intervention studies: safety, tolerability, and dosing

Current clinical development of urolithin A is still centered on oral nutritional supplementation rather than conventional drug-style dose escalation. From a regulatory perspective, the U.S. FDA has already issued a “no questions” response to GRAS Notice No. 791 for specified food uses of urolithin A, which supports a basic safety framework for intended food-use exposure but does not establish disease-specific efficacy (80). In interventional development, current randomized studies are testing 500 mg/day and 1,000 mg/day regimens for up to 6 months in healthy middle-aged adults (81), supplementation in frail older adults with outcomes centered on muscle mitochondrial quality (82), and 500 mg/day monotherapy as well as lower-dose combination strategies in middle-aged and older adults with sleep- and aging-related endpoints (83). Other registered trials are evaluating effects on glucose metabolism, insulin secretion, incretin responses, and mitochondrial function in adults aged 55 years and older (84), as well as vascular, cerebrovascular, nitric oxide-related, and mitochondrial endpoints in middle-aged adults with obesity (85). At the formulation level, dedicated single-dose pharmacokinetic studies are also comparing seven and four Mitopure formulations in healthy adults, incorporating concentration-time profiling, exposure metrics, and tolerability monitoring directly into the trial design (86, 87). These studies collectively suggest that the human safety and dosing literature is growing, but it is still relatively fragmented across populations, formulations, and endpoint frameworks. The principal human studies are summarized in Table 3.

### 5.2 Clinical endpoints and biomarkers of target engagement

A notable feature of the current clinical pipeline is the shift from broad wellness-oriented claims toward more mechanism-linked endpoints. ATLAS 2 is designed around muscle strength and performance outcomes in healthy middle-aged adults (81), whereas the frail older adults trial focuses more directly on mitochondrial quality in skeletal muscle (82). The sleep-focused randomized study includes aging-biomarker readouts rather than relying only on subjective wellbeing measures (83). The glucose-metabolism trial extends this target-engagement strategy into insulin secretion, incretin biology, and mitochondrial function in brain and muscle tissues (84). Similarly, the obesity-associated vascular dysfunction protocol centers on endothelial function, cerebrovascular regulation, nitric oxide bioavailability, and mitochondrial biomarkers (85). Together, these studies indicate that the field is increasingly attempting to align human endpoints with the mechanistic pathways most often implicated in pre-clinical studies, especially mitochondrial quality control, metabolic function, and vascular physiology.

### 5.3 Why pre-clinical and clinical findings diverge

Several factors help explain why robust pre-clinical mechanisms do not consistently translate into clear clinical efficacy. First, exposure constraints matter: physiologically based pharmacokinetic modeling predicts that circulating and tissue concentrations achievable with supplementation are substantially lower than many concentrations used in cell systems, even though they remain well below predicted toxic levels (31). Second, formulation itself is a development variable rather than a trivial formulation detail. Ongoing comparative pharmacokinetic studies of multiple Mitopure preparations suggest that exposure profiles cannot simply be assumed to be identical across products (86, 87). Third, the dominant human circulating species after dietary exposure are often conjugated rather than free aglycone forms. After 4 weeks of pecan consumption in humans, plasma urolithins were reported mainly as glucuronide conjugates (88). Taken together, these observations help explain why aglycone-based *in vitro* studies or supraphysiologic exposure paradigms may overestimate *in vivo* relevance, and why nominally similar human supplementation studies may still differ in target engagement. Beyond these mechanistic considerations, the current clinical evidence base itself has important methodological limitations that constrain firm efficacy conclusions. Most completed trials are small, of short duration (typically a few weeks to a few months), and powered for biomarker or surrogate endpoints rather than hard functional or clinical outcomes; several report neutral results on their primary functional endpoints despite favorable biomarker changes (7, 8, 57). Endpoint selection is heterogeneous across studies, follow-up is rarely long enough to capture durable functional benefit, and many trials are conducted in healthy or relatively homogeneous populations, limiting both statistical power and generalizability. These limitations should be weighed explicitly when interpreting the apparent promise of urolithin A.

### 5.4 Responder heterogeneity, metabotypes, and trial design challenges

Responder heterogeneity remains one of the central translational challenges in this field. In a randomized clinical trial of pomegranate supplementation in overweight-obese adults, clustering by urolithin metabotype explained interindividual variability in cardiovascular risk-marker improvement, with lipid benefits emerging mainly in specific metabotype-defined subgroups (89). This finding is important because it shows that host response to ellagitannin-rich interventions cannot be interpreted independently of microbial conversion phenotype. It also implies that studies based on dietary precursors and studies based on direct urolithin A supplementation should not be treated as interchangeable models of exposure or responsiveness (86, 87, 89). More recent trial development also suggests a move toward broader phenotype enrichment, including cognition- and brain-fog-related outcomes in adults using Mitopure-containing products (90). Even so, the field still lacks a standardized strategy for prospectively stratifying participants by metabotype, exposure phenotype, or tissue-level target engagement, which remains a key reason why pre-clinical and clinical findings can diverge in magnitude or consistency. Importantly, metabotype-based stratification has its own limitations that must be acknowledged when used for trial design. Metabotype assignment can show temporal instability within individuals, is sensitive to the amount and pattern of precursor intake and to broader dietary background, and depends on methodological choices such as classification thresholds, sampling matrices (urine vs. plasma vs. feces), and analytical windows (3, 4, 36). Consequently, a single cross-sectional classification may misrepresent an individual's habitual conversion capacity, and harmonized, prospectively validated criteria are needed before metabotyping can be reliably embedded in clinical stratification.

## 6 Toward clinical application: precision nutrition and therapeutic development

### 6.1 Metabotype-guided precision nutrition

A clinically meaningful application of urolithin science will likely require moving from uniform supplementation strategies toward metabotype-guided precision nutrition. In a *post-hoc* analysis of the MaPLE trial, older adults classified by urolithin metabotype showed differential responses to a polyphenol-rich dietary intervention, with UM-B participants exhibiting a twofold greater improvement in zonulin than UM-A participants (91). This finding is important because it suggests that urolithin metabotypes are not merely descriptive metabolic phenotypes, but may also predict the magnitude and direction of host response to diet-based interventions (91). More recently, a broader metabotype-clustering study integrating ellagic acid, resveratrol, and daidzein metabolism showed that urolithin-related phenotypes coexist with other microbial-response traits in distinct clusters, each associated with different gut microbiome structures and functional networks (92). Accordingly, future precision-nutrition strategies may need to classify participants not only as UM-A, UM-B, or UM-0, but also within more composite microbiome-metabolic strata that better reflect real-world interindividual variability. Translating metabotype-guided nutrition into routine clinical practice will require addressing several practical implementation issues. These include the availability and turnaround of validated metabotyping assays, agreement on sampling matrix and classification thresholds, integration of phenotype results into dietary or supplementation decisions, and consideration of cost and accessibility. In practice, a stratified pathway can be envisaged in which converters are guided toward ellagitannin-rich dietary patterns while non-converters are directed to defined supplementation or microbiome-targeted strategies, with response monitored through exposure biomarkers rather than precursor intake alone. Realizing this pathway depends on the standardization and prospective validation steps discussed above and in Section 7.

### 6.2 Enhancing endogenous urolithin a production through diet and microbiome modulation

One translational route is to enhance endogenous urolithin generation by selecting diets and food matrices that favor both precursor delivery and a permissive microbial ecology. Short-term walnut consumption has already been shown to modulate gut microbiota differently according to baseline urolithin metabotype, with UM-B-associated microbiota appearing more responsive to the intervention (93). In mild dyslipidaemic overweight-obese individuals, pomegranate intake also altered bile acid and cholesterol metabolism in a way that correlated quantitatively with fecal urolithin concentrations, particularly urolithin A, suggesting that endogenous production may itself act as a driver of downstream metabolic effects rather than merely as a passive exposure marker (94). These observations support the idea that dietary modulation should not be framed only as “providing ellagitannins,” but rather as shaping a microbiome–substrate system capable of producing relevant downstream metabolites. Supporting evidence also comes from a controlled supplementation study in the SHIME model, in which pomegranate extract altered microbial composition and metabolite production across colonic compartments (95). Even so, current data also indicate that food-first strategies are likely to produce variable responses, and that the efficiency of endogenous production will depend on both the composition of the intervention and the baseline metabolic competence of the host microbiome.

### 6.3 Microbiome-targeted strategies

A second, more interventionist path is to target the microbiome directly. *In vitro* fermentation work using human intestinal microbiota from UM-A donors showed that ellagic acid-to-urolithin A conversion was associated with higher abundances of Bifidobacterium longum, Bifidobacterium adolescentis, and Bifidobacterium bifidum, nominating these taxa as potential contributors to the conversion ecosystem (96). Although these data do not yet establish causality at the single-strain level, they help define a mechanistic basis for developing microbiome-targeted approaches that go beyond descriptive metabotyping. Proof-of-concept work is also beginning to test whether urolithin production can be enhanced by administering urolithin-producing bacteria themselves. In mice, oral supplementation with *Gordonibacter urolithinfaciens* promoted ellagic acid metabolism and increased urolithin bioavailability, while synbiotic delivery also enriched taxa such as Akkermansia, Lactobacillus, and Bifidobacterium (97). A recent updated review has consequently framed urolithin-producing strains as candidate next-generation probiotics and highlighted screening methods, fermentation optimization, metabolic-pathway analysis, and synthetic-biology approaches as key tools for future development (98). At present, however, this field remains pre-clinical, and major questions remain regarding colonization durability, interspecies interactions, scalability, and safety evaluation in humans.

### 6.4 Direct supplementation vs. endogenous formation

Direct supplementation and endogenous formation should not be viewed as interchangeable exposure models. In mice, repeated ellagic acid administration resulted in detectable ellagic acid in feces but not in plasma or liver, and urolithin A was not detected across those samples, whereas direct urolithin A administration generated measurable downstream conjugated metabolites (99). This comparison underscores a central translational point: supplying precursors does not guarantee meaningful systemic urolithin exposure, because both intestinal transformation and host absorption remain limiting steps. Quantitatively, this gap is substantial: after dietary precursor intake, circulating free ellagic acid rarely exceeds ~100 nm and is highly variable between individuals, and urolithins appear in plasma only after ~24 h and pre-dominantly as conjugates, with high inter-individual variability driven by metabotype (13, 15, 16). In low-conversion individuals (UM-0), dietary precursors may yield little or no measurable systemic urolithin exposure at all, whereas a single direct dose of urolithin A produces severalfold higher and far more consistent plasma exposure across the population (7, 26). Thus the disparity between administration routes is not merely qualitative but spans orders of magnitude in achievable systemic exposure, and is widest precisely in the populations least able to convert precursors.

By contrast, direct supplementation offers a defined and standardized molecule. The European Commission novel food application describes supplemental urolithin A as a synthetic ingredient identical in structure to the compound formed endogenously after ellagitannin and ellagic acid intake, manufactured to a well-defined high-purity specification and micronized to improve homogeneity (100). In practical terms, endogenous formation preserves the broader diet–microbiome interaction and may capture additional ecological effects of ellagitannin-rich foods, whereas direct supplementation is better suited to clinical programs that require predictable exposure, controlled dosing, and reduced dependence on baseline microbial conversion capacity. These considerations support a practical decision framework. Endogenous biogenesis can reasonably be prioritized when the goal is dietary prevention in individuals with demonstrated conversion capacity (UM-A or UM-B), where ellagitannin-rich foods deliver both urolithins and the wider matrix of co-occurring nutrients. Direct supplementation becomes effectively indispensable when predictable, therapeutic-range exposure is required—for example in clinical trials with defined endpoints, or in non-converter (UM-0) and low-converter individuals who fall below the minimum microbial transformation threshold needed to generate bioactive urolithins. For such non-converter populations, alternative strategies include direct supplementation with the defined molecule or, prospectively, microbiome-directed approaches (targeted probiotics, defined consortia, or pre-biotic conditioning) aimed at installing or enhancing conversion capacity, although the latter remain investigational (26).

### 6.5 Formulation and delivery strategies

Formulation is likely to become a decisive factor as urolithin A moves closer to clinical application. The Australian Therapeutic Goods Administration has already published a compositional guideline for urolithin A permitted for use in listed medicines, specifying identity testing, assay thresholds, purity limits, residual-solvent limits, and elemental-impurity criteria (101). This is important because future interventional studies will need to distinguish clearly between nominally similar “urolithin A” products that may differ in manufacturing quality, physicochemical properties, and exposure behavior.

Delivery-system development is also accelerating. A pH-driven liposomal formulation improved the stability, bioaccessibility, and oral bioavailability of urolithin A, achieving approximately 1.9-fold higher bioavailability than a traditional thin-film liposomal preparation in rats (102). In a separate study, PEGylated urolithin A liposomes showed improved storage stability, sustained release, enhanced cellular uptake, and higher pharmacokinetic exposure than free urolithin A solution, including increases in half-life and area under the curve (103). These data suggest that future formulation strategies may need to be matched to therapeutic intent—for example, maximizing systemic exposure, prolonging circulation time, or favoring tissue-specific delivery—rather than assuming that the free compound is always the optimal translational form.

## 7 Safety, standardization, and regulatory development

### 7.1 Toxicological and long-term safety considerations

The current safety case for urolithin A is stronger at the pre-clinical toxicology level than at the level of long-term human use. In a dedicated safety assessment of directly administered synthetic urolithin A, the compound was reported to be non-genotoxic, with glucuronidated and sulfonated metabolites pre-dominating in ADME studies, and no adverse effects observed in 28-day or 90-day oral studies in rats; the NOAEL was the highest dose tested (104). These data provide an important toxicological foundation because they evaluate the administered molecule itself rather than relying only on historical dietary exposure to ellagitannin-rich foods.

However, long-term safety in humans remains less mature than the pre-clinical package. For example, the completed MitoImmune study enrolled 50 healthy adults and was designed to test whether oral urolithin A can modulate mitochondrial activity in immune cells and improve immune function, illustrating that the human development program is still centered mainly on relatively short, mechanistic intervention studies rather than prolonged real-world exposure in clinically complex populations (105). Therefore, the available evidence is more consistent with a conclusion of acceptable short-term to subchronic safety than with a fully resolved long-term safety profile across diverse patient groups; this is an inference based on the present balance of toxicology and clinical-development data. The boundaries of this extrapolation should therefore be stated explicitly. Current NOAEL values derive from rodent 28- and 90-day studies, and the available human safety data come pre-dominantly from short-term trials in healthy adults; neither directly addresses long-term, continuous use in metabolically vulnerable populations (104, 105). Patients with metabolic disease, hepatic or renal impairment, polypharmacy, or altered phase II conjugation capacity, as well as the elderly and individuals using the product chronically, fall outside the populations in which safety has actually been characterized. Long-term safety in these groups should be regarded as not yet established, and dedicated studies in representative populations and exposure durations are needed before such extrapolation is justified.

### 7.2 Standardization of products, exposure, and outcome measures

As urolithin A moves toward broader clinical application, standardization must extend beyond simple nominal dose reporting. At the product level, a recent validated UHPLC method was developed specifically for determining urolithin A content in health products, explicitly noting that the lack of standardized and reliable quantification methods has been a major gap for quality control and regulatory compliance (106). This point is highly relevant for translational work because inconsistent assay methods can obscure whether different products labeled as urolithin A are actually comparable in content and purity. Exposure standardization is equally important. The completed Bioavailability of Mitopure Formulations study was designed to compare concentration-time profiles across five formulations in a controlled single-dose setting, underscoring that formulation-dependent pharmacokinetics must be treated as part of the intervention rather than as a minor technical detail (107). Accordingly, future studies should standardize at least three linked domains: product identity and assay, formulation-specific exposure metrics, and outcome measures that reflect target engagement rather than relying only on broad clinical readouts. It should be emphasized that the core challenge of standardization is not the availability of analytical methods *per se*, but the comparability of exposure data generated by different detection systems and under different formulation conditions. Even where validated assays exist, results obtained on different LC–MS/MS platforms, in different biological matrices, or for different conjugated species are not directly comparable unless harmonized against common reference standards and reporting conventions. Formulation compounds this problem: liposomal, micronized, and free-form preparations can differ substantially in AUC, Cmax, and metabolite profiles, so that nominally identical doses produce non-equivalent systemic exposure. This formulation-dependent variability is a major and currently under-emphasized source of inconsistency across clinical studies, and should be reported as an integral part of the intervention rather than as a technical footnote.

### 7.3 Regulatory and clinical development considerations

Recent regulatory documents show that urolithin A is being evaluated within increasingly formalized novel-food and product-authorization frameworks. In the United Kingdom, the ACNFP first review of the urolithin A application identified information gaps across identity, production process, composition, stability, specifications, ADME, and toxicology (108). A subsequent ACNFP discussion paper then framed the key regulatory question more explicitly: whether the available data are sufficient to conclude that urolithin A is safe and not nutritionally disadvantageous under the proposed uses and use levels (109). These documents indicate that regulatory review is focused not only on toxicology, but also on dossier completeness and internal consistency across chemistry, manufacturing, exposure, and safety sections. A similar trajectory is visible in continental Europe. In Switzerland, the FSVO determined that urolithin A meets the definition of a novel food and is therefore subject to authorization requirements (110). More broadly, the EU framework provides that only novel foods authorized and included in the Union list may be placed on the market (111), and EFSA's updated guidance specifies that applications must document the description of the novel food, production process, compositional data, specifications, proposed uses and use levels, and anticipated intake (112). Together, these developments suggest that future clinical development of urolithin A will depend not only on demonstrating biological activity, but also on meeting increasingly explicit requirements for identity, exposure characterization, safety substantiation, and fit-for-purpose endpoint selection. Beyond describing these frameworks, it is important to recognize their scientific constraining effect on how research is conducted. By requiring unambiguous compound identification, defined specifications, traceable exposure characterization, and internally consistent safety and efficacy dossiers, regulators such as the ACNFP and EFSA are effectively driving the field away from the simple verification of a single active ingredient and toward a standardized system built on clear identity, traceable exposure, and unified study endpoints. In this sense, regulatory requirements are not merely administrative hurdles but are actively shaping study design, analytical standardization, and endpoint selection in urolithin research.

## 8 Knowledge gaps and future directions

### 8.1 Defining the true active species *in vivo*

One of the most important unresolved questions in the field is what the true active species actually is *in vivo*. Biological activity may not be attributable to a single pool of free urolithin A alone, but instead to a dynamic mixture that can include unconjugated aglycones, glucuronidated and sulfated conjugates, transient upstream intermediates, and possibly locally deconjugated forms in inflamed or metabolically altered tissues (12, 113). This uncertainty is not merely conceptual; it reflects the fact that urolithins show complex metabolic distribution across biological compartments and that rigorous chromatographic and spectroscopic discrimination of structurally related metabolites remains analytically demanding. Future studies should therefore move beyond binary statements such as urolithin A was detected and instead define exposure using chemically resolved metabolite panels across plasma, urine, feces, and, where feasible, tissue compartments.

### 8.2 Establishing causal microbes and metabolic pathways

A second major gap is the transition from descriptive metabotypes to causal microbial and enzymatic mechanisms. Recent work has substantially advanced the field by identifying a dehydroxylase encoded by human gut Enterocloster species that enables diet-derived urolithin A formation, thereby moving one key step of the pathway from correlation toward molecular causation (114). Additional work has shown that the presence and induction of regioselective dehydroxylases differ across Enterocloster species, helping to explain why related strains can generate distinct urolithin products and why cross-feeding and pathway branching are likely to matter *in vivo* (115). Even so, human intervention data still remain partly correlative: in a walnut dietary study, multiple bacterial genera and inferred microbial functions were associated with distinct urinary urolithin-metabolite patterns, but these associations do not yet fully establish which microbes are necessary, sufficient, or context-dependent for specific pathway steps (116). Accordingly, future research needs integrated strain isolation, functional genetics, transcriptomics, and ecosystem-level validation rather than further reliance on metabotype labels alone.

### 8.3 Standardizing exposure assessment and clinical endpoints

Another priority is the standardization of both exposure assessment and clinical endpoints. Analytical work has already shown that meaningful exposure characterization requires more than measuring a single nominal urolithin A signal, because multiple structurally related metabolites and conjugates can coexist in biological samples and require careful separation and identification (113). More recent assay development has demonstrated that multi-metabolite urinary panels are technically feasible, including validated quantification of nine urolithin metabolites in human urine (117). On the clinical side, future studies should pre-specify which matrix is primary, whether free or total analytes are being measured, what sampling window defines exposure, and which endpoints truly represent target engagement rather than non-specific downstream effects. Biomarker programs should also be validated in a fit-for-purpose, context-of-use framework if trial results are to become comparable across products, populations, and indications (118).

### 8.4 Expanding beyond urolithin A

The field also needs to expand beyond its current urolithin A-centric focus. Although urolithin A has dominated mechanistic and translational studies, newer evidence indicates that other urolithins are not biologically redundant. Urolithin C has been reported to suppress colorectal cancer progression through AKT/mTOR-related signaling (37), and separate work suggests that urolithin C can also suppress inflammatory signaling in activated macrophages through NF-κB-related mechanisms (38). In parallel, urolithin D is now being investigated as a potentially relevant oxidative-stress-modulating metabolite (39). These findings do not yet establish non-urolithin-A species as clinically superior alternatives, but they do show that the broader urolithin family may encode distinct structure–activity relationships that remain underexplored. Future work should therefore compare multiple urolithins head-to-head under matched exposure conditions rather than assuming that all meaningful biology converges on urolithin A alone.

### 8.5 Integrating urolithins into precision nutrition and precision medicine

Ultimately, integrating urolithins into precision nutrition and precision medicine will require linking microbial conversion capacity, validated exposure biomarkers, and indication-specific outcome measures within a single clinical framework. A 12-week randomized controlled trial has already shown that a metabotype-based personalized nutrition strategy can improve dietary quality and selected metabolic-health parameters relative to population-level advice (119). More broadly, metabotyping has been proposed as a practical approach for identifying clinically relevant nutrition-response subgroups, although larger trials and stronger implementation frameworks are still needed before routine application (120). Recent perspectives also argue that personalized nutrition is moving away from one-size-fits-all models toward multi-omic and context-aware strategies (121), while broader discussions in the field emphasize that phenotyping-driven personalization must also be embedded within real-world dietary, behavioral, and sustainability constraints (122). For urolithins, this means that future clinical application will likely depend on matching intervention mode—dietary precursors, microbiome modulation, or direct supplementation—to each individual's metabolic competence, microbiome ecology, and disease context.

## 9 Conclusions

Urolithins have emerged as a compelling interface between diet, gut microbiota, host metabolism, and age-related disease biology. Across the literature reviewed in this manuscript, the field has progressed from identifying urolithins as gut microbial metabolites of ellagitannins and ellagic acid to recognizing them as biologically active compounds with potential relevance to mitochondrial quality control, inflammation, metabolic regulation, tissue resilience, and healthy aging. Among the currently studied molecules, urolithin A has received the greatest mechanistic and translational attention and therefore serves as the principal reference point for evaluating the broader clinical significance of this metabolite family. At the mechanistic level, one of the most influential concepts has been the ability of urolithin A to promote mitophagy and improve mitochondrial health. This framework has helped unify findings across multiple disease contexts, including skeletal-muscle aging, metabolic dysfunction, neuroinflammation, cardiovascular injury, intestinal barrier impairment, and immune aging. At the same time, the evidence reviewed here also shows that urolithin biology cannot be reduced to mitophagy alone. Anti-inflammatory signaling, redox regulation, lipid metabolism, epithelial-barrier effects, and microbiome-dependent biotransformation all contribute to the observed phenotype and together shape the translational relevance of these compounds. A major strength of the field is that it now spans the full continuum from microbial ecology and metabolic biotransformation to pre-clinical disease models and early human intervention studies. However, this breadth has also exposed an important translational gap. Pre-clinical findings are often robust and mechanistically rich, whereas human studies remain more limited in size, duration, standardization, and endpoint consistency. In particular, differences in exposure biology, formulation, circulating metabolite species, host microbiome composition, and baseline metabolic competence complicate direct comparison between experimental systems and clinical outcomes. As a result, promising mechanistic plausibility does not yet equate to established clinical efficacy across specific diseases. Another key conclusion is that interindividual variability is not a secondary issue but a defining feature of urolithin research. The capacity to generate urolithins from dietary precursors differs markedly across individuals, and this variability appears to influence both exposure and physiological response. The concepts of urolithin metabotypes and responder heterogeneity therefore have major implications for both nutrition research and therapeutic development. Future applications will likely depend on whether interventions aim to enhance endogenous production through food and microbiome modulation or to bypass metabolic variability through direct supplementation with standardized urolithin preparations. The reviewed evidence also indicates that the field should move beyond an exclusive focus on urolithin A. Other metabolites, including urolithin B, urolithin C, isourolithin A, and related compounds, may have distinct biological properties that are not fully captured by an urolithin A-centered framework. Expanding comparative studies across the broader urolithin family may therefore reveal new structure-activity relationships, new therapeutic opportunities, and a more complete understanding of how host and microbial metabolism jointly shape biological function. From a translational perspective, the next phase of the field will require greater rigor in several areas. These include defining the true active species *in vivo*, identifying causal microbes and metabolic enzymes, standardizing exposure assessment, harmonizing clinical endpoints, improving formulation and delivery strategies, and integrating biomarker-based target engagement into trial design. Just as importantly, regulatory development will need to advance alongside scientific discovery, with clearer standards for product identity, purity, safety substantiation, and clinically meaningful claims.

Overall, urolithins represent one of the most promising examples of how gut microbiota-derived metabolites can be translated into precision nutrition and possibly precision-medicine strategies. Yet their future impact will depend less on repeating the general claim that they are “beneficial” and more on defining for whom, under what exposure conditions, through which metabolite species, and for which clinical indications they are most effective. In this sense, the urolithin field is moving from descriptive enthusiasm toward a more mature phase of mechanism-based, stratified, and clinically testable development.
